# The past, present, and future of endoscopic management for biliary strictures: technological innovations and stent advancements

**DOI:** 10.3389/fmed.2024.1334154

**Published:** 2024-11-28

**Authors:** Dong-jin Ni, Qi-fan Yang, Lu Nie, Jian Xu, Si-zhe He, Jun Yao

**Affiliations:** ^1^Department of Gastroenterology, The Affiliated People’s Hospital of Jiangsu University, Zhenjiang, China; ^2^Department of Intervention Vascular, Wujin Hospital Affiliated with Jiangsu University, Changzhou, China; ^3^Shanghai Academy of Fine Arts, Shanghai University, Shanghai, China

**Keywords:** biliary stricture, endoscopic treatment, biliary stents, biodegradable materials, precision medicine

## Abstract

Biliary stricture can be induced by intrinsic narrowing and extrinsic compression, with the majority of cases being malignant. Clinically, distinguishing between benign and malignant biliary strictures remains a considerable challenge, and the ongoing disagreement over the optimal choice of biliary stents significantly influences treatment strategies and impacts patients’ survival and prognosis. The utilization and advancement of endoscopic techniques have heightened the diagnostic sensitivity for biliary strictures. Concurrently, innovative technologies such as endoscopic ultrasound and magnetic compression anastomosis emerge as viable alternatives when endoscopic retrograde cholangiopancreatography (ERCP) is not an option, providing fresh insights for the clinical management of these patients. Traditional plastic and metal stents, characterized by their complex application and limited scope, have been unable to fully satisfy clinical needs. The introduction of novel stent varieties has notably improved this scenario, marking a considerable progression towards precision medicine. However, the clinical validation of the diverse stent materials available is incomplete. Hence, a thorough discussion on the present state and evolving trends of biliary stents is warranted.

## 1 Introduction

**Figure 1:**
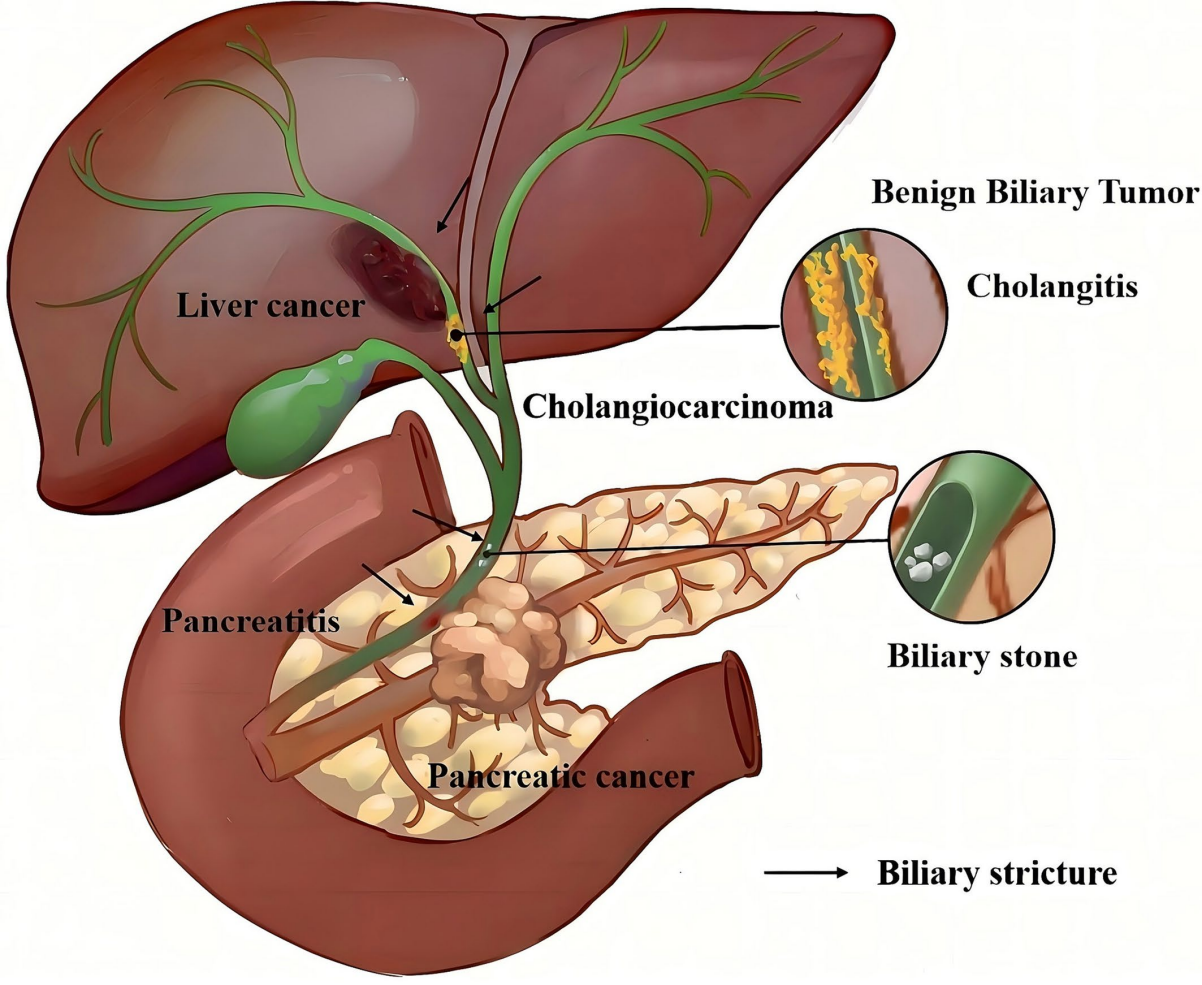
Hepatobiliary system and partly causes of biliary strictures.

**Figure 2:**
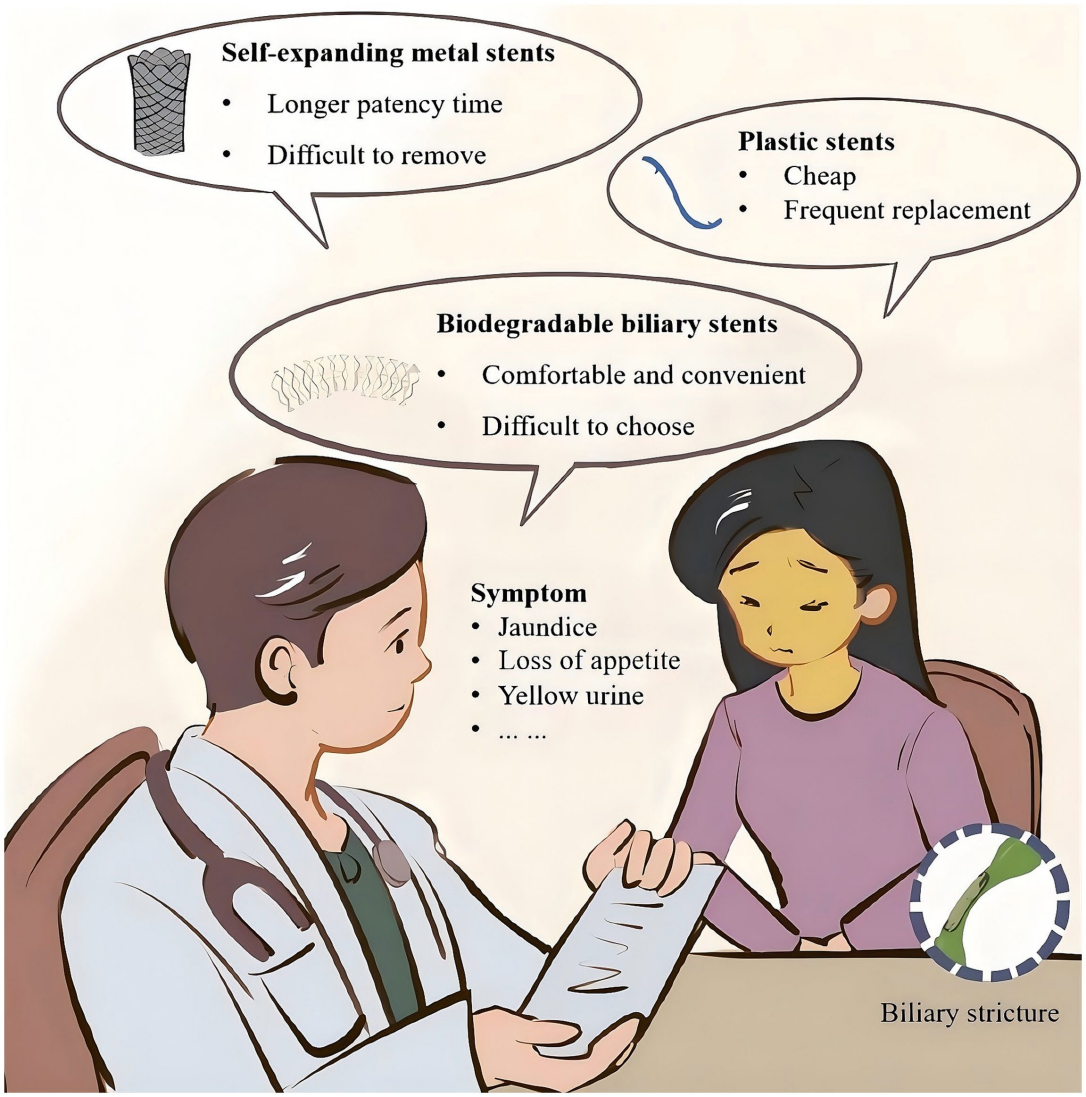
Symptoms of patients with biliary strictures and stents treatment.

The biliary system functions as the main channel for hepatocytes to convey bile, crucial for the digestion of fats and fat-soluble vitamins. Pathological factors, including trauma, surgical interventions, inflammation, or tumors, can lead to biliary strictures or complete obstruction (Figure 1). This results in bile accumulation and subsequent clinical manifestations such as jaundice, pruritus, and urine discoloration (Figure 2) (1). Persistent biliary blockage can bring complications such as ascending cholangitis, Gram-negative sepsis, and liver abscesses, presenting substantial health and mortality risks (2). Research has indicated that 74–87% of patients with biliary strictures suspected to be malignant diagnosed by endoscopic retrograde cholangiopancreatography (ERCP) or endoscopic ultrasound (EUS) are found to have malignancies, and indeterminate biliary strictures are more likely to be malignant (3). However, differentiating between benign biliary strictures (BBS) and malignant biliary strictures (MBS) continues to be a significant clinical hurdle, markedly affecting therapeutic strategies and patient outcomes. The intricate structure of the bile duct, coupled with its narrow lumen-whether in the intrahepatic bile duct or the common bile duct-makes it challenging to obtain direct histological evidence of an intrinsic stricture, even when its location is pinpointed (4). Additionally, lesions in adjacent organs like the liver and pancreas can influence the bile duct’s patency, adding layers of complexity to the diagnostic process. Furthermore, conditions such as primary sclerosing cholangitis and autoimmune pancreatitis inherently obscure the distinction between benign and malignant manifestations (5, 6). Therefore, discerning the cause of biliary stricture, whether BBS or MBS, continues to be a significant medical challenge. In the past, percutaneous surgery was commonly used to manage biliary stricture. However, as an invasive procedure, it is evident that it causes more discomfort to the patients. Additionally, studies have shown that it has a higher mortality rate compared to endoscopic treatment in the management of malignant hilar biliary strictures (7). Medical technology advancements have established endoscopic diagnosis and treatment as the foremost clinical approach for biliary strictures, owing to its minimal invasiveness and procedural simplicity (3, 8). Effective management of BBS and MBS centers on alleviating the constriction to facilitate bile drainage. The emergence of biliary stents has enabled extended drainage, but the selection between plastic stents (PS) and self-expanding metal stents (SEMS) continues to be debated. Currently, the American Gastroenterological Association (AGA) and the European Society of Gastrointestinal Endoscopy (ESGE) providing relevant recommendations in its updated guidelines (3, 9). The emergence of novel biliary stents has expanded therapeutic options for biliary strictures. This review presents recent advancements in their diagnosis and treatment, emphasizes the features and latest trends of various innovative biliary stents, and provides a fresh perspective on the clinical management of biliary strictures.

## 2 Etiology and classification of biliary stricture

Based on causative factors, they can be categorized into BBS and MBS. Various non-neoplastic factors, including iatrogenic injuries and inflammatory lesions, can lead to these strictures. Notably, iatrogenic strictures represent the predominant cause, with post-cholecystectomy patients (whether laparoscopic or open) being the most commonly affected (3, 9). Biliary strictures following cholecystectomy frequently affect the common hepatic duct and the proximal common bile duct. The etiology of these strictures include undue traction on the gallbladder neck during surgery, injury from electrocautery, and fibrosis resulting from local inflammation of the cystic duct (9, 10). Biliary strictures following liver transplantation also rank among the prevalent causes of biliary stricture (11, 12). It occurs more frequently in living donor liver transplantation compared to deceased donor liver transplantation (13, 14). Secondly, inflammatory lesions rank as the second leading cause of benign biliary strictures (2). Examples include pancreatitis and cholangitis stemming from various origins: acute pancreatitis, chronic pancreatitis (inclusive of autoimmune pancreatitis), and primary sclerosing cholangitis (15, 16). Additionally, other benign causes of biliary strictures, such as autoimmune inflammatory strictures and benign tumors, are listed in Table 1. MBS are caused by primary or secondary tumors. Primary cholangiocarcinoma is a common cause of malignant biliary strictures, as it grows or spreads directly within the bile ducts, leading to narrowing or complete obstruction (17). Moreover, malignant tumors of neighboring organs, such as pancreatic cancer and gallbladder cancer, can infiltrate or compress the bile ducts, causing strictures (18). Secondary MBS may be caused by metastasis from cancers in other parts of the body. Detailed causes of MBS are provided in Table 1.

**Table 1:** Causes of biliary strictures.

Category	Cause	Specific descriptions
BBS	Iatrogenic injury	Post-cholecystectomy, post-liver transplantation, post-endoscopic sphincterotomy, post-other cholangioenterostomy
	Autoimmune inflammation	IG4 sclerosing cholangitis, autoimmune pancreatitis, primary sclerosing cholangitis
	Non-autoimmune inflammation	Acute pancreatitis, chronic pancreatitis, acute suppurative cholangitis, inflammatory pseudotumor
	Benign tumors	Bile duct adenoma, giant cell tumor, papillary tumors, intraductal papillary neoplasm of the bile duct
	Obstructive	Mirizzi syndrome, portal biliary disease, common bile duct stone
	Infectious diseases	HIV infection, parasites, biliary tuberculosis
	Ischemic diseases	Post-intrahepatic perfusion of chemotherapy drugs, hepatic artery stenosis or embolism, vasculitis
MBS	Primary tumors	Cholangiocarcinoma, pancreatic cancer, hepatocellular carcinoma, ampullary carcinoma, gallbladder carcinoma
	Secondary tumors	Metastatic tumors from other sites, lymphoma, metastasis from peripheral lymph nodes

BBS, benign biliary strictures; MBS malignant biliary strictures.

## 3 Diagnosis and treatment of endoscopic biliary strictures

### 3.1 Diagnosis

Endoscopic diagnosis stands as the paramount method for discerning the property of biliary strictures. However biopsy/brush cytology conducted by ERCP exhibits constrained sensitivity. A meta-analysis of 9 studies indicated that the combined sensitivity of cytology brushings and intraductal biopsies under ERCP for diagnosing malignant biliary strictures was 45 and 48.1%, respectively (19). Endoscopic ultrasound-guided fine needle aspiration/biopsy (EUS-FNA/FNB) has an 80% sensitivity for malignant biliary strictures. However, it has challenges in accessing proximal biliary strictures and the risk of tumor dissemination along the FNA channel, which could potentially lead to cancer spread (20). This technique should be used with caution when addressing intrahepatic biliary strictures or post-liver transplantation biliary strictures. Consequently, diagnosing malignant biliary strictures remains a focal point of research. Leveraging varied endoscopic techniques, several innovative detection methods have emerged to enhance sensitivity. Peroral cholangioscopy (POC) and probe-based confocal laser endomicroscopy (pCLE) are among the most sensitive approaches. POC is a technique for direct imaging of the biliary tract. It allows the endoscopist to insert a scope into the bile ducts for visualization. This method enhances specimen collection, with studies showing that adequate pathological specimens can be obtained in 94.4% of patients (21). One limitation of EUS is the difficulty in accessing proximal bile duct stenosis, as well as concerns that malignant tumors may disseminate along the FNA channel. However, A research study has reviewed the effectiveness of peroral cholangioscopy (POC) in diagnosing biliary strictures, emphasizing its advantages in detecting proximal lesions (22). Probe-based confocal laser endomicroscopy (pCLE) combines the functionalities of optical microscopy and endoscopy. By inserting an optical probe through the cholangioscope, it enables the acquisition of real-time, high-resolution histological images, which significantly aids in differentiating proximal lesions (23). Han’s et al. (24) research included 59 patients with primary sclerosing cholangitis and reported a diagnostic sensitivity of 85.7% for pCLE, with the highest sensitivity observed at the bifurcation and right hepatic duct. However, limitations in pCLE probe technology may reduce diagnostic sensitivity for the common bile duct and left hepatic duct.

Some new technologies can also increase the detection rate of MBS. Centrifuge bile samples collected from ERCP or EUS to separate potential cancer cells. Subsequently, fix the sediment and embed it in paraffin to form a cell block similar to a tissue nted this stent into the bile ducts of rabbits, which not only reduced bacterial adhesion but also minimized tissue proliferation. With the advancement of machine learning, artificial intelligence has demonstrated unique advantages in diagnosing MBS. Convolutional neural networks (CNN), a type of deep learning model, are particularly suited for processing grid-structured data such as images and videos. CNN can enhance low-quality images and achieve automatic image diagnosis by analyzing these images. Additionally, their diagnostic capabilities improve as the sample size increases (25). While these novel diagnostic methods offer promise, they come with challenges such as elevated costs, operational complexities, and restricted accessibility. Future advancements in diagnosing malignant biliary strictures may build upon current technologies, mitigating technical and operational challenges. A feasible approach is to use clinical prediction models or various machine learning algorithms to enhance the identification of high-risk patients, thereby reducing the additional financial burden on them.

### 3.2 Endoscopic therapy

#### 3.2.1 Biliary drainage under ERCP

Stent insertion via ERCP and endoscopic nasobiliary drainage (ENBD) facilitates bile drainage and alleviates biliary obstruction. Notably, the combination of ERCP with biliary stent placement remains the primary approach for addressing biliary strictures. Contemporary stent varieties encompass PS, SEMS and other novel stents (9). Traditionally, PS have been suitable for all biliary stricture categories, with frequent utilization of multiple stent placements (MPS) to alleviate obstructions. While several SEMS types exist, they are predominantly employed for extrahepatic biliary strictures. However, research offers divergent perspectives on the indications for distinct metal stents. For example, in a meta-analysis encompassing five multicenter randomized controlled studies with 781 global participants, Saleem et al. (26) showed that fully covered self-expanding metal stents (FCSEMS) outperformed uncovered self-expanding metal stents (UCSEMS) in treating distal MBS and had longer patency time. Conversely, a multicenter randomized controlled trial conducted by Conio et al. (27) indicated that when treating extrahepatic MBS, FCSEMS experienced increased stent migrations and earlier stent occlusions compared to UCSEMS.

When comparing SEMS to PS, initially considering cost, no notable difference was observed between the two for patients with a survival duration exceeding 1 year or falling below 3 months (28). Secondly, regarding efficacy, the overall performance of SEMS and PS in treating BBS is analogous. However, the choice of stent varies based on the cause and location of the stricture. The European Society of Gastrointestinal Endoscopy (ESGE) recommends the endoscopic placement of self-expanding metal stents (SEMS) primarily for the treatment of malignant extrahepatic biliary obstruction (9, 28, 29). Both stent varieties present notable limitations. PS is susceptible to obstruction, and repeated ERCP stent replacements introduce increased surgical risks and challenges (30, 31). The terminal portion of the SEMS stent may injure the intestinal or bile duct wall, while prolonged stent placement can result in bile sludge accumulation and stone development (32). ESGE advises that the therapeutic benchmark for BBS involves placing MPS or SEMS. For inoperable MBS patients, SEMS is the first choice (9). There remains a lack of unanimous agreement regarding the utilization of various SEMS types, necessitating further prospective studies. Additionally, other novel stents unable to gain widespread clinical adoption, and their therapeutic effectiveness warrants further investigation and validation. Furthermore, ENBD serves as an alternative for bile drainage. The nasobiliary tube, introduced via the nasal cavity into the bile duct, facilitates the drainage of accumulated bile, alleviating obstruction. Kawashima’s et al. (33) study indicates that ENBD can effectively reduce patients’ total bilirubin levels, improve the prognosis of patients (34). Compared to percutaneous transhepatic biliary drainage (PTBD), endoscopic nasobiliary drainage (ENBD) has a lower risk of tumor spread following surgery (35, 36). However, in patients with proximal or intrahepatic bile duct obstruction, PTBD provides more complete drainage, shorter drainage times, and better recovery of liver function compared to endoscopic retrograde biliary drainage (ERBD) (37). Numerous studies have highlighted the advantages of PTBD in cases of advanced hepatic portal stenosis (38). Additionally, long-term use of a nasobiliary duct following ENBD may lead to laryngeal irritation, water and electrolyte imbalances, and risks such as rupture, detachment, or displacement. Therefore, ENBD is typically utilized for short-term symptom relief rather than for long-term treatment.

#### 3.2.2 Biliary drainage under EUS

Approximately 5–10% of patients cannot successfully undergo ERCP (9, 39). Common complete obstructions of the bile duct in these patients are due to distal MBS or postoperative anatomical changes in BBS, making it difficult for an endoscope to pass through the duodenal papilla (40, 41). For individuals unsuitable for ERCP drainage, conventional treatments encompass PTBD drainage or endoscopic intervention. However, these methods carry an elevated risk of complications and extended hospitalizations (42, 43). Endoscopic ultrasound-guided biliary drainage (EUS-BD) presents a minimally invasive and safer option, appropriate for patients unsuitable for ERCP biliary drainage (44). EUS-BD can be performed through stent placement and fistula creation for drainage. Endoscopic ultrasound-guided choledochoduodenostomy (EUS-CD) or hepaticogastrostomy (EUS-HG) is typically chosen. EUS-CD is suitable for distal bile duct strictures, while EUS-HG is appropriate for gastric outlet obstruction or postoperative bile duct strictures. The practitioner can initially position an ultrasound probe within the stomach or duodenal bulb to facilitate ultrasonic imaging of the bile duct. Subsequently, utilizing a needle, they access the intrahepatic bile duct or common bile duct and introduce contrast. Ultimately, under needle guidance, a stent is positioned to facilitate bile drainage into the stomach or intestinal cavity, alleviating biliary obstruction (45, 46). EUS allows for direct puncture into the bile duct through the stomach or duodenal wall, thereby avoiding the duodenal papilla.

Compared to PTBD, EUS-BD is not only more effective in patients with MBS but also has fewer adverse events and complications, with a lower rate of reintervention (47–49). Importantly, for patients with inoperable malignant biliary strictures who have failed ERCP-guided biliary stent placement, endoscopic ultrasound-guided biliary drainage (EUS-BD) appears to be a superior treatment option. EUS-BD is associated with a higher success rate for biliary drainage and fewer complications compared to percutaneous transhepatic biliary drainage (PTBD) (49, 50). In comparison with ERCP, EUS-BD boasts a comparable success rate and, on the whole, a diminished likelihood of adverse events. In specific scenarios, like patients experiencing biliary obstruction post-gastroduodenal stent placement, EUS-BD might exhibit superior technical success (51). With the ongoing advancement of the EUS-BD technique, research is now delving into identifying patients who might benefit more from EUS-BD than ERCP, or even positioning EUS-BD as the principal drainage approach for those not suitable for surgery (52–54). However, numerous EUS-BD procedures exist, each with intricate indications. Consequently, further research is imperative to elucidate the risks and advantages of each procedure and ascertain its efficacy across diverse biliary stricture types.

#### 3.2.3 Other novel endoscopic techniques

Techniques such as endoscopic radiofrequency ablation (RFA), ^125^I particle implantation, and magnetic compression anastomosis (MCA) are progressively integrated into biliary stricture treatment protocols. RFA is frequently employed for the palliative care of MBS. By generating electromagnetic waves via high-frequency alternating current, RFA induces intense heat and coagulative necrosis in the stenotic tissue, markedly enlarging the constricted bile duct’s diameter (32, 55). Likewise, ^125^I particle implantation serves as a therapeutic approach for MBS. ^125^I particles, being a low-energy radiation source, can inflict direct harm to the DNA double helix structure. This disrupts tumor cell replication, induces cell apoptosis, and potentially activates CD3^+^ and CD4^+^ cells, eliciting an anti-tumor immune response (56). ^125^I seeds can be attached to the stent and inserted into the bile duct, providing targeted destruction of the tumor tissue. Employing endoscopic ultrasound-guided ^125^I particle in conjunction with stent implantation markedly enhances the survival rate and quality of life for MBS patients, exhibiting a robust safety profile (57, 58). Traditionally, this therapeutic strategy is predominantly employed through the PTC approach. Regarding the endoscopic method, comprehensive research is essential to elucidate the operational technique, application range, efficacy, and safety of ^125^I particle implantation. It is noteworthy that for patients presenting with complete biliary stricture or those unresponsive to ERCP and percutaneous liver puncture therapy, MCA stands as a viable option (59). MCA employs regional compression between two magnets to proficiently accomplish the recanalization of complete stenoses, thereby reinstating normal drainage at the stenotic site through the induction of ischemic necrosis and epithelialization within the affected area. Studies indicate that the recanalization success rate of MCA ranges between 77 and 100% (60–62). During treatment, physicians typically employ endoscopic or percutaneous techniques to position magnets at both ends of the bile duct stricture—one at the proximal end and the other at the distal end. As the magnets attract each other within the body, the tissue at the stricture site becomes compressed and undergoes necrosis. This process allows the magnets to gradually converge, ultimately restoring the patency of the bile duct. While these emerging technologies hold potential as alternatives to conventional drainage methods, but a standardized criterion for assessing their application techniques and indications is unable to be established. Consequently, the selection of an appropriate technology remains reliant on the clinical acumen and technical expertise of practicing physicians.

## 4 Evolution of biliary stents

### 4.1 Plastic stents

PS pioneered clinical usage as a biliary stent. Initiated by Professor Soehendra from Germany, who first employed a plastic stent for the endoscopic treatment of distal common biliary strictures in 1979, endoscopic stent placement has become a prevalent practice in clinical treatments (63). Typical materials for plastic stents encompass polyethylene, polyurethane, and polytetrafluoroethylene (Teflon). Owing to its superior flexibility and strength, polyethylene remains the predominant choice in clinical settings (64). Teflon exhibits a higher possibility to perforation compared to polyethylene, whereas polyurethane demonstrates diminished strength, potentially sustaining damage during removal (65–67). The prevalent plastic biliary stents vary in length from 1 to 18 cm and feature a range of diameters from 5 to 12F, encompassing both straight and pigtail types. Due to their cost-effectiveness, ease of removal compared to SEMS, and safety, they find extensive utilization in clinical settings. The guidelines of ACG advocate for prioritizing PS placement in patients with benign biliary strictures (BBS) who retain the gallbladder, as well as in cases of perihilar biliary stricture induced by cholangiocarcinoma (3). Furthermore, BBS patients utilizing PS should undergo treatment for a duration exceeding 12 months. Intestinal bacteria may adhere to the stent’s inner surface via the stent opening, fostering the formation of bacterial biofilms. This phenomenon renders plastic stents susceptible to blockages (68). Firstly, the predominant solution involves multiple stent replacements. Research indicates that substituting plastic stents with progressively increasing diameters can more effectively facilitate tissue remodeling (69). A significant drawback of this approach is the necessity for recurrent endoscopic examinations, necessitating at least three annual visits, which might result in patients disregarding medical advice and exacerbating the discomfort associated with treatment. Secondly, drug-coated novel stents can effectively reduce the adhesion of various bacteria, but there is still a lack of research to evaluate the impact on systemic drug concentrations after stent placement (70). Lastly, employing an anti-reflux plastic stent (ARPS) presents a viable alternative to counter the infiltration of intestinal bacteria. Dua et al. (71) pioneered the use of ARPS in 2007, demonstrating that this stent could prolong the median patency duration from 101 to 145 days. Conversely, Vihervaara et al. (72) undertook a clinical study utilizing the identical ARPS, but their study was prematurely halted owing to early stent occlusions within the ARPS group. This group exhibited a median stent patency of merely 34 days, markedly less than the 167 days observed in the general stent group. Therefore, while theoretically ARPS has the potential to diminish the incursion of duodenal bacteria and enhance stent patency, further comprehensive studies are requisite to substantiate its clinical efficacy.

### 4.2 Self-expanding metal stents

In 1990, Gillams pioneered the use of UCSEMS in the treatment of benign biliary strictures (73). SEMS, an expandable mesh structure, can be initially positioned at sizes of 8.5F or smaller (74). Upon expansion, it attains a diameter that substantially surpasses that of PS (24–30F). Regarding materials, SEMS typically incorporate elements such as platinum, characterized by a platinum core enveloped in a nickel-titanium alloy shell, stainless steel, or nickel-titanium alloy (75). Clinically, three prevalent SEMS models are utilized: UCSEMS, FCSEMS, and partially covered self-expanding metal stents (PCSEMS). According to ACG, a confirmed diagnosis of MBS is requisite prior to the utilization of UCSEMS (3). Moreover, patients undergoing treatment with FCSEMS for BBS should adhere to a treatment duration of up to 6 months. In instances where the treatment extends beyond 12 months, a stent replacement is mandated at the 6-month mark. Once positioned, UCSEMS tends to remain stable, resisting displacement (76). However, it exhibits a relatively high incidence of stent occlusion, attributed to tissue ingrowth within the stent. Furthermore, its removal post-placement presents a considerable challenge. PCSEMS features a central coating within the stent, allowing the ends to embed within the tissue, thereby mitigating the risk of stent displacement. While it facilitates short-term endoscopic removal, it is associated with a higher incidence of severe adverse events, particularly migration (77, 78). The pharmaceutical coating adorning the surface of FCSEMS can mitigate tissue ingrowth, albeit augmenting the likelihood of stent displacement or slippage. Furthermore, exacerbated tissue proliferation at the stent extremities, coupled with sludge accumulation, can precipitate stent obstruction (27, 79). Currently, a myriad of novel stents are under development to enhance clinical efficacy. Cho et al. (80) successfully minimized tissue proliferation and sludge accumulation by infusing nickel-titanium stents with nanosilver particles. Park et al. (81) devised a novel spiral spring biliary metal stent capable of significantly reducing the displacement rate while concurrently delaying occlusion. The main limitation of FCSEMS is frequent displacement and removal complications. Park et al. (82) incorporated fixed winglets at the proximal end of FCSEMS or adopted an outward design at the same location, a modification that substantially diminishes the migration rate following stent placement. Regarding the selection of SEMS, despite the scrutiny in preceding studies, the findings pertaining to patency, safety, and cost-effectiveness remain disparate (75). Consequently, a definitive clinical standard governing its utilization is unable to be established. The clinical applicability of PS and SEMS still needs to be based on their advantages and disadvantages and the clinical characteristics of the patients, combined with the experience of clinical doctors and surgical conditions. For more details, please refer to Table 2.

**Table 2:** Comparisons of PS and SEMS.

Type	Advantages	Disadvantages	Clinical applications	References
SEMS FCSEMS PCSEMS	FCSEMS can prevent mucosal growth and tissue invasion	FCSEMS is prone to displacement	Long-term drainage	(3, 10, 30, 66, 74)
UCSEMS	UCSEMS can be embedded in the bile duct and is not easily displaced	UCSEMS is prone to blockage and difficult to remove after placement	Palliative treatment for MBS patients less than 12 months	
	Larger lumen, stronger support	May lead to complications like cholecystitis and pancreatitis	Preoperative biliary drainage for MBS	
	Longer patency time (12 months)	Tumor ingrowth	FCSEMS treatment for BBS should be more than 6 months, and if more than 12 months, the stent should be replaced at 6 months	
	No need for multiple treatments	High initial cost	UCSEMS treatment should be based on a clear diagnosis of MBS	
PS	Low cost	Prone to displacement	Long-term treatment for BBS patients	(3, 30, 66, 67)
	Easy to insert and remove	Shorter patency time (3–6 months)	MBS patients with a survival time of more than 12 months	
	Smaller lumen, multiple stents can be inserted at once	Needs frequent replacement, increasing patient discomfort	Patients with preserved gallbladder	
	High safety for long-term placement	May lead to cholangitis, stone formation, etc.	Temporary drainage for undiagnosed patients	
	Wide applicability, more flexible use	Overall cost comparable to SEMS		

PS, plastic stents; SEMS, self-expanding metal stents.

### 4.3 Biodegradable biliary stents

Biodegradable biliary stents (BDBS) have emerged as a novel type of biliary stent. In tandem with the progression of medical technology and evolving clinical demands, their research and implementation have progressively garnered attention. BDBS exhibit superior biocompatibility and undergo natural degradation within the body, thereby obviating complications associated with prolonged stent placement. This feature also precludes the necessity for subsequent surgical intervention for stent removal, markedly enhancing the patient’s quality of life and the facilitation of treatment (1, 83–86). The primary materials for BDBS include polyesters and magnesium-based alloys. It can also serve as a drug carrier to treat diseases or delay obstruction (1, 74). A comparison of the characteristics of various materials and their research status can be found in Table 3.

**Table 3:** Comparisons of material features and research status of BDBS stents.

Material category	Features	Research status	Degradation time	References
PDX	Commonly used, good flexibility, low mechanical strength	Human (*in vivo*)	3–6 months	(82, 106)
PLA	Good mechanical properties, adjustable degradation time, self-cleaning	Pigs, dogs, *in vitro* (bile)	>9 months	(1, 94, 95)
PCL	Good biocompatibility, drug-controlled release, low melting point	Pigs, *in vitro* (bile)	3 months	(96, 97, 102, 104)
PGA	Good biocompatibility, fast degradation	Pigs	2 months	(106, 108)
PLGA	Fast but adjustable degradation	*In vitro* (bile)	2–3 weeks	(90)
PTMC	Average mechanical strength, adjustable degradation time	Rats, *in vitro*	10–14 weeks	(109, 110)
Magnesium alloy	Widely used, safety, adjustable degradation speed, can inhibit the development of biliary tumors	Rabbits	20 weeks	(115–117)

PDX, polydioxanone; PLA, polylactic acid; PCL, polycaprolactone; PGA, polyglycolic acid; PLGA, poly(lactic-co-glycolic acid); PTMC, poly (trimethylene carbonate).

#### 4.3.1 Polyester material

##### 4.3.1.1 Polydioxanone

Polydioxanone (PDX) as a preeminent biodegradable material, widely used in clinical fields such as bio-sutures, coronary stents, and biliary stents (1). It is fabricated from the dioxanone monomer, undergoing degradation into glycolic acid via hydrolysis of its ester bonds. Presently, its application has permeated the domain of biliary stents. PDX stents exhibit exceptional flexibility and mechanical properties. These stents are capable of undergoing natural degradation over a period of 3–6 months, thereby obviating the requirement for subsequent surgical intervention for removal (84, 87). This development markedly amplifies the quality of life for patients and facilitates the treatment process. Regarding efficacy, Siiki et al. (87) orchestrated a prospective study on 13 BBS patients and 83% of them did not require further intervention after the placement of a PDX stent (single stent insertion, length 40–80 mm, diameter 8–10 mm). Mauri et al. (85) conducted a two-year surveillance on 107 patients who underwent PDX stent (diameter: 8–10 mm, length: 40–70 mm. Customized per patient’s condition) implantation, noting a patency rate surpassing 80% (no further invasive treatment was needed). Pertaining to safety, Siiki’s et al. (87) study highlighted that 25% of patients encountered mild cholangitis during the stent’s placement, while 17% experienced restenosis, potentially attributable to the stent’s gradual degradation and possible rupture. Giménez et al. (88) implanted PDX biliary stents (10 mm in diameter by 40 mm long) in 13 patients and followed up for 21 months; aside from one case of restenosis and one of cholangitis, 84.6% of the patients symptom-free. Anderloni’s et al. (89) prospective study involving 38 patients indicated that the migration rate of PDX stents was analogous to that of FCSEMS. Considering design, PDX stents exhibit potential for further refinement. Huang et al. (90) scrutinized the performance and impacts of 12 distinct biliary stent structures, analyzing the fluid and structural interactions among bile, bile ducts, and polydioxanone biliary stents. Consequently, they devised two superior stent model structures to rectify the existing structural deficiencies. In conclusion, the preliminary validation of the clinical efficacy and safety of PDX stents has unveiled promising clinical prospects. However, additional development and research are imperative to enhance the stent design and facilitate larger-scale prospective clinical studies for evaluating efficacy and potential complications.

##### 4.3.1.2 Polylactic acid

While the safety and efficacy of PDX have garnered initial verification, its short degradation time remains a significant limitation. Conversely, Polylactic acid (PLA) exhibits excellent mechanical attributes in bile, undergoing a degradation process that spans beyond 9 months (91). Its characteristics can be modulated by amalgamating different materials, bestowing PLA with considerable versatility in medical applications (92). In recent years, PLA has found extensive utilization in the fabrication of biodegradable bone screws, bone plates, vascular stents, and tissue engineering scaffolds (1, 93–95). Furthermore, PLA stents have demonstrated possibility in non-vascular lumens, catalyzing their adoption in the domain of biliary stents. Zhang et al. (96) implanted PLA and PDX stents in porcine bile ducts, observing that the PLA stents exhibited a prolonged deformation period (23 weeks compared to 11 weeks) and an extended median patency duration (25.7 weeks versus 11.3 weeks), without any notable postoperative complications in either group. Yamamoto et al. (91) implanted PLA stents in canine bile ducts, noting the onset of degradation between 6 to 9 months. A fraction of 22% (2 out of 9) of the stents became embedded in the bile duct wall, with no complications documented across all experimental subjects. Remarkably, the PLA material exhibits self-cleaning properties. Meng et al. (97) immersed PLA films in human bile for a duration of 2 months. Starting from the third week, the surface of the PLA samples began to degrade, diminishing the accumulation of bile sludge, thereby highlighting the promising self-cleaning attributes of PLA. Meanwhile, the PE stent used in the experiment showed no significant effect. However, despite the numerous benefits associated with PLA, its application in human subjects remains unexplored, and animal studies are limited. Comprehensive evaluations of its safety, efficacy, and biocompatibility necessitate further extensive animal experiments and clinical trials for validation.

##### 4.3.1.3 Polycaprolactone

Polycaprolactone (PCL) constitutes an outstanding biodegradable polyester substance characterized by minimal toxicity and optimal biocompatibility (98). However, its low melting point (Tm 57°C) restricts its utilization as an independent stent material, predominantly serving as a coating substance for other stents at present (99–102). Hu and Lin (103) employed PCL as a medium to incorporate silver nanoparticles (AgNPs) and cisplatin (DDP). Utilizing electrospinning, they fabricated a PCL-AgNPs-DDP fibrous-coated dual-functional airway stent, demonstrating efficacy in diminishing microbial adhesion and granulation tissue proliferation. Kim et al. (104) illustrated that a stent, which integrates PCL with a sorafenib drug-eluting component, significantly curtails angiogenesis, proliferation, and invasion of tumor cells in mice afflicted with bile duct cancer. Jang et al. (105) fabricated a dual-layer drug-eluting stent (DES) by integrating a 3D-printed paclitaxel-PCL stent with SEMS, effectively curtailing the proliferation of malignant tumors in patients suffering from hilar MBS. Moreover, PCL stents preserve remarkable ductility and flexibility post-implantation. Oh et al. (106) demonstrated *in vitro* that the PCL stent maintained over 80% of its initial radial force even after 15 weeks and sustained more than 90% of its initial strength following 56 days. In contrast, the PDX stent experienced a reduction of over 50% in its mechanical strength within a span of 14 days. Consequently, PCL demonstrates a brighter potential for application in the treatment of BBS compared to PDX. The shortcomings of PCL stents are quite apparent. Kim et al. (104) inserted stents primarily composed of PCL into the bile ducts of pigs, observing that 27.3% (3/11) of the stents were displaced, and an equal percentage experienced stent fractures. Moreover, the fibrosis thickness in the bile duct of the stent group, measured at 0.46 mm, was significantly greater than that of the control group, which was 0.21 mm. However, the fibrosis thickness observed in this study remains within a relatively safe margin, not inducing complications such as biliary obstruction or bile leakage in the pigs, thereby illustrating its promising safety and feasibility. The worrying thing is this study lasted only 3 months and did not evaluate efficacy. Therefore, longer-term studies are needed to assess its safety (whether the degree of fibrosis will cause new strictures) and effectiveness (whether it can reduce obstructions in the long run). In conclusion, PCL exhibits more pronounced benefits compared to other polyester materials. While it has demonstrated promising clinical efficacy and safety as a coating material, further investigations concerning its technological application and design are warranted when utilized as the main component in stent fabrication.

##### 4.3.1.4 Other polyester materials

Besides the materials previously mentioned, other biodegradable substances also demonstrate significant potential in the realm of biliary stents. Polyglycolic acid (PGA) is a singular component material characterized by favorable biocompatibility, currently finding applications in bone implants, tracheal stents, and various other domains (107). However, its mechanical strength is somewhat limited, sustaining merely for a period of 2 weeks in bile. Furthermore, it undergoes rapid degradation, being completely absorbed within a mere 2 months, which restricts its utility in the fabrication of biliary stents (108). Kwon et al. (108) implanted PGA stents in the bile ducts of pigs, observing a swift decline in the stents’ mechanical properties beginning from the second week, with noticeable degradation and deformation commencing by the sixth week. The degradation byproducts did not induce any severe adverse reactions, initially affirming its safety for use as a biliary stent. In a similar vein, the degradation period of Poly (lactic-co-glycolic acid) (PLGA) in bile spans 2–4 weeks, with its mechanical attributes sustaining merely for 4 days (109). PLGA is synthesized through the polymerization of lactic acid and glycolic acid monomers. Jan et al. (110) suggested that PLGA exhibits superior biocompatibility and facilitates controlled drug release. Encapsulation within the cell membrane can prolong the circulation duration of PLGA nanoparticles within the organism, enhancing targeting capabilities and minimizing systemic toxicity, thus positioning it as a promising material for drug delivery. Zeng et al. (92) manipulated the proportion of PLGA polymers and discovered that the 80/20 PLGA composition exhibits a slower degradation rate, preserving its mechanical properties for an extended period. This suggests its potential as a primary material for BDBS, although additional research and validation are requisite. Furthermore, poly (trimethylene carbonate) (PTMC) exhibits considerable promise. PTMC represents a novel category of aliphatic polycarbonate biodegradable substances, extensively utilized in bone implants and ureteral stents (111). However, PTMC has not been applied in the bile ducts. Zheng et al. (112) also noted in their study that while the mechanical strength of PTMC is somewhat inferior, this limitation can be mitigated through polymerization with other substances. In conclusion, despite the nascent stage of research concerning these materials in the domain of biliary stents, their prospective potential should not be underestimated.

#### 4.3.2 Magnesium alloy

Apart from polyester substances, magnesium (Mg) has demonstrated considerable potential in the realm of biodegradable materials, attributed to its an excellent biocompatibility (113). Through the analysis of its degradation characteristics in bone and blood environments, it has found extensive applications in orthopedic materials and cardiovascular stents (114, 115). Similarly, Mg has exhibited potential in the realm of biliary stents. Liu et al. (116) revealed that the weight reduction of the magnesium alloy WE43 did not surpass 18% within a span of 60 days, having the potential to become a biliary stent. Regarding biocompatibility within the bile duct, Song et al. (117) introduced magnesium alloy AZ31 into the bile ducts of rabbits, routinely monitoring pertinent indicators such as bilirubin and hemoglobin. The findings indicated that these markers remained stable within the normal range throughout the duration of the study. Furthermore, by the 20th week, the AZ31 stent had fully degraded, and the concentrations of magnesium and zinc ions generated through degradation did not surpass safe thresholds. These observations suggested that the AZ31 magnesium alloy possesses favorable biocompatibility and safety within the bile duct. Besides its potential as a material for BDBS, magnesium inherently exhibits a certain inhibitory effect on gallbladder cancer. Li et al. (118) illustrated in their study that extracts of magnesium have the potential to curb the growth of human cholangiocarcinoma cells, fostering their apoptosis, in addition to hindering tumor cell adhesion and the synthesis of cytoskeletal proteins. Peng et al. (119) injected gallbladder cancer cells subcutaneously into nude mice, establishing subcutaneous xenograft tumors, followed by the insertion of magnesium wires into these subcutaneous tumors. Following a 24-day period, both the tumor volume and weight in the group with magnesium wire implantation exhibited a notable reduction compared to the control group, illustrating that magnesium possesses a significant inhibitory effect on the proliferation of gallbladder cancer tumors. Currently, magnesium alloy biliary stents have gradually been put into clinical use. Magnesium alloy stents have exhibited multifaceted efficacy in bile duct drainage and anti-tumor activities, holding substantial promise in the management of benign and malignant biliary strictures. However, comprehensive large-scale prospective clinical trials are imperative to corroborate their clinical effectiveness in human biliary applications.

### 4.4 Future trend stents

#### 4.4.1 Drug eluting stents

DES are a new type of stent developed based on existing stents. These stents are endowed with coatings that facilitate the controlled release of medicinal agents within the organism, thereby curtailing tumor proliferation or mitigating inflammatory responses, consequently realizing therapeutic outcomes (74). Firstly, the antineoplastic agents sanctioned by the FDA constitute the predominant categories of DES, including prominent examples such as paclitaxel (PTX) and gemcitabine (GEM). PTX has garnered extensive utilization in cardiovascular stents, exhibiting the capacity to forestall restenosis through the inhibition of fibroblast activity and collagen metabolism (120). The application of PTX in the bile duct is under investigation. Tao (121) demonstrated that the paclitaxel-N-succinyl hydroxyethyl chitosan sustained-release film effectively curtails scar formation in the bile ducts of rabbits. Jang et al. (122) amalgamated sodium hexanoate with PTX to augment the localized anti-tumor effects, a strategy that not only markedly decelerated the constriction of the pig’s bile duct but also prolonged the time until stent obstruction. Xiao et al. (123) implanted a mixed DES of GEM and cisplatin in the pig’s bile duct. This approach not only curtailed tumor proliferation but also exhibited a notable safety profile (no complications in any group). Secondly, Antibiotic-coated DES have demonstrated promising potential. Rapamycin can forestall biliary stricture through the inhibition of fibroblasts, while gentamicin can restrain intestinal flora, thereby diminishing the onset of cholangitis and postponing stent obstruction (70, 124). Furthermore, mitomycin-C curtails RNA synthesis and decreases protein expression, consequently reducing the prevalence of scar formation within the bile duct (125). DES has exhibited substantial therapeutic efficacy and potential. Various drug coatings are formulated to target distinct primary ailments, thereby facilitating enhanced treatment outcomes and presenting considerable versatility in the clinical management of biliary stricture.

#### 4.4.2 3D printing stent

3D printing technology amalgamates computer-aided design, material processing, and computer-aided manufacturing methodologies, facilitating enhanced design adaptability in stent fabrication. This innovation permits personalization according to patient-specific requirements, enabling the realization of intricate shapes and affording precise control over dimensional attributes (1, 126). 3D printed stents have been initially utilized in domains such as cardiovascular, tracheal, and esophageal interventions (127). Thomas et al. (128) used tissue engineering techniques to create an extrahepatic bile duct model with mechanical properties similar to those of the biliary tract. Boyer et al. (127) pioneered the application of this technology in the biliary sector, fabricating a 3D printed stent for the bile duct utilizing cross-linked polyethylene glycol. While it has not been transitioned to clinical application, this development establishes a cornerstone for the creation of patient-specific stent manufacturing technology. Kim et al. (104) developed a biodegradable 3D printed biliary stent, which exhibited promising safety and feasibility in pig models (no complications or adverse events). In addition, 3D printing technology can be combined with BDBS and DES, allowing the customization of stents based on the characteristics of the patient’s bile ducts and the use of different materials according to the disease. Lee et al. (129) designed a 3D-printed stent using PCL, with a surface coated with zinc ions and sirolimus. They implanted this stent into the bile ducts of rabbits, which not only reduced bacterial adhesion but also minimized tissue proliferation and sludge formation. However, the implementation of 3D printing technology necessitates the involvement of specialized equipment and skilled personnel, and further investigation and refinement of its biocompatibility and mechanical attributes are imperative. In conclusion, 3D printing technology heralds novel avenues in the design and fabrication of biliary stents, marking a substantial progression in personalized and precision medical interventions.

#### 4.4.3 Tissue engineering stents

Tissue-engineered stents are novel stents that mimic the extracellular matrix in the body to promote the formation of new tissue and the restoration of function. In the wake of the relentless advancements in biomaterial science and tissue engineering technology, both stem cells and induced pluripotent stem cells can be guided to differentiate into biliary cell (130). Furthermore, cholangiocyte organoids hold a significant advantage in simulating physiological microenvironments (131). Leveraging this technology, stents enveloped with living tissue surfaces have been engineered, facilitating integration with patient physiology or serving as a viable option for biliary reconstruction to mend the bile duct, thereby demonstrating promising potential in the management of biliary stricture (132). Boyer et al. (127) conceptualized a novel approach involving the utilization of 3D printing technology amalgamated with collagen injection molding to fabricate bio-integrated biliary stents. This methodology encompasses the infusion of collagen, human placental mesenchymal stem cells, and biliary cells, thereby affirming the viability of these innovative stents. Biodegradable materials, when synergized with tissue engineering techniques, exhibit superior biocompatibility (133). Li et al. (134) utilized a PCL stent as a scaffold to facilitate the proliferation, migration, and differentiation of biliary cells. This research substantiated that the PCL stent fosters cell proliferation on its surface, thereby serving as a viable framework for the development of biologically active artificial bile ducts. Zong et al. (135) fabricated a dual-layer stent utilizing PCL and PLGA, incorporating human bone marrow mesenchymal stem cells, and subsequently implanted them into 18 pigs. Within a span of 6 months, none of the experimental animals exhibited any indications of biliary stricture or bile stasis. Moreover, the tissue-engineered stent demonstrated superior reparative effects on biliary injuries compared to the blank PCL/PLGA stent. This suggested that tissue-engineered stents represent a novel methodology for addressing biliary stricture. These stents can be tailored to meet individual patient needs, boasting undeniable biocompatibility. Moreover, they facilitate the rapid regeneration of biliary endothelial cells, thereby expediting the repair process of the bile duct. Despite being in the nascent stages of development, tissue-engineered stents have exhibited encouraging initial outcomes, hastening bile duct recovery and reducing the duration of treatment cycles. However, this type of stent is currently in the animal testing stage, comprehensive evaluations of their long-term safety and efficacy are necessary to be conducted.

## 5 Conclusion and outlook

The advancement in endoscopic treatment for biliary stricture has been substantial, with the utilization of PS and SEMS markedly enhancing the alleviation of patients’ clinical symptoms and improving post-treatment outcomes. However, given the intricate etiology of biliary stricture, along with pronounced individual variations and differing patient survival durations, the two existing stent types retain certain limitations in their therapeutic efficacy. Firstly, a majority of patients are required to undergo numerous endoscopic procedures to facilitate the removal and reinsertion of stents, thereby escalating both the physical discomfort and financial burden. Consequently, this could deter patients from adhering to regular medical consultations, potentially compromising the efficacy of the treatment (69). Secondly, due to the complex course of the bile duct, stent displacement is a problem. Once displaced, additional surgery is required to address the issue. Although UCSEMS can be fixed by tissue ingrowth after insertion, its irretrievability makes its clinical application more cautious, and only patients with a clear diagnosis are suitable (3). Therefore, enhancing the post-treatment quality of life for patients and making personalized selections based on comprehensive clinical evaluations continue to be paramount. The forthcoming generation of stents presents promising avenues to mitigate these existing challenges. On the one hand, there should be continued research and development of biodegradable materials with superior mechanical properties and biocompatibility, regulating degradation time, and perfecting BDBS design. On the one hand, there should be continued research and development of biodegradable materials with superior mechanical properties and biocompatibility, regulating degradation time, and perfecting BDBS design. On the other hand, 3D printing and tissue engineering technologies can be used to customize biliary stents for patients, providing more precise individualized treatment based on their conditions. These innovative technologies have the potential to significantly mitigate the discomfort associated with secondary surgeries and reduce postoperative complications. Furthermore, DES facilitates treatment adjustments based on the root causes, theoretically augmenting the likelihood of favorable clinical outcomes. However, the existing research remains nascent. Although good physicochemical properties and safety have been demonstrated in animal and *in vitro* experiments, only a few studies have been applied to humans. Therefore, clinical efficacy has not been widely recognized. Moreover, the diverse characteristics of various stent materials, although adaptable to complex conditions, pose a challenge to the clinical practitioner’s accurate assessment of the condition. This necessitates not only interdisciplinary collaboration between medical and other fields, but also an amplification of prospective clinical research efforts to substantiate their efficacy. Such advancements are poised to mitigate patients’ distress, enhance their quality of life, and extend the benefits of medical advancements to a broader patient population.
